# Association of dysphagia with altered brain glucose metabolism in Parkinson's disease

**DOI:** 10.1111/cns.14214

**Published:** 2023-04-11

**Authors:** Ji Yeon Oh, Eui Jin An, Young Lee, Seung Min Kim, Miju Cheon, Jun Yup Kim

**Affiliations:** ^1^ Department of Physical Medicine and Rehabilitation Veterans Health Service Medical Center Seoul South Korea; ^2^ Veterans Medical Research Institute Veterans Health Service Medical Center Seoul South Korea; ^3^ Department of Neurology Veterans Health Service Medical Center Seoul South Korea; ^4^ Department of Nuclear Medicine Veterans Health Service Medical Center Seoul South Korea; ^5^ Department of Physical Medicine and Rehabilitation Hanyang University Medical Center Seoul South Korea

**Keywords:** brain glucose metabolism, dysphagia, hypermetabolism, hypometabolism, Parkinson's disease

## Abstract

**Aims:**

Dysphagia is a major clinical concern in Parkinson's disease (PD). However, the relationship between the development of phase‐specific dysphagia and the regional brain glucose metabolism remains unclear. Our objective was to investigate the distributions of brain glucose metabolism specific to oral and pharyngeal phases of dysphagia in PD.

**Methods:**

In this retrospective cross‐sectional study, patients with PD who underwent videofluoroscopic swallowing study (VFSS) and ^18^F‐fluorodeoxy‐glucose positron emission tomography at intervals of <1 month were included. Each swallow was assessed by the binarized Videofluoroscopic Dysphagia Scale with 14 subitems, seven each for the oral and pharyngeal phases. Metabolism mapping was performed by superimposing significant clusters of subitems belonging to each of the two phases using voxel‐wise Firth's penalized binary logistic regression model, adjusting for age and PD duration at VFSS.

**Results:**

Eighty‐two patients with PD who met the inclusion criteria were included in the analysis. The oral phase dysphagia‐specific overlap map showed hypermetabolism in the right inferior temporal gyrus, bilateral cerebellum, superior frontal gyrus, and anterior cingulate cortices. Hypometabolism in the bilateral orbital and triangular parts of the inferior to middle frontal gyrus was also correlated with the occurrence of oral phase dysphagia. The development of pharyngeal phase dysphagia was related to hypermetabolism of posterior aspects of the bilateral parietal lobes, cerebellum, and hypometabolism of the mediodorsal aspects of anterior cingulate and middle to superior frontal gyri.

**Conclusion:**

These findings suggest that phase‐specific distribution of brain glucose metabolism may explain the dysphagia of PD.

## Graphical abstract

Metabolic mapping using subitem scores from video fluoroscopic swallowing studies and ^18^F‐fluoro‐2‐deoxy‐d‐glucose positron emission tomography in patients with Parkinson's disease revealed altered distributions in cerebral glucose metabolism specific to oral and pharyngeal dysphagia, respectively. Our findings suggest potential targets of symptom‐specific transcranial neuromodulation for dysphagia in Parkinson's disease.

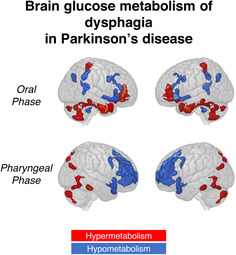

## 1 INTRODUCTION

Digestive system disorders result in a significant deterioration in the quality of life and increase the medical expenses of patients with Parkinson's disease (PD), and these include gastroesophageal reflux, nausea, epigastric bloating, constipation, and dysphagia.^1^, ^2^ Of these, dysphagia can lead to malnutrition and pneumonia, posing a serious threat to health and maintenance of life, and up to 90% of patients with PD eventually develop dysphagia.^3^, ^4^

Neuroanatomical areas responsible for normal human swallowing process include the cerebellum, thalamus, insula, lateral pre‐ and postcentral gyri, superior temporal gyrus, middle and inferior frontal gyri, frontal operculum, occipital gyrus, precuneus, and anterior cingulate cortex.^5^, ^6^ Aside from the normal swallowing, dysphagia has also been shown to occur by lesions of other cerebral regions such as inferior frontal gyrus, supramarginal gyrus, angular gyrus, insula, thalamus, putamen, globus pallidus, and amygdala.^7^, ^8^, ^9^ Furthermore, several studies have reported the effect of targeted transcranial stimulation on dysphagia‐related neuroanatomical regions after stroke^10^, ^11^; however, despite the significant debilitating effects of dysphagia in patients with PD, relationship between the altered regional glucose metabolism of brain and development of dysphagia has not yet been elucidated. When the distribution of glucose metabolism related to each subitem of dysphagia in PD is identified, the effects of targeted transcranial stimulation on dysphagia symptoms can be tested via stimulating the corresponding cortical area, similar to that of noninvasive neuromodulatory stimulation targeted to other motor symptoms in PD.^12^, ^13^

Cerebral metabolic distributions by ^18^F‐fluoro‐2‐deoxy‐d‐glucose positron emission tomography (^18^F‐FDG‐PET) for each specific symptom of PD (e.g., akinetic‐rigid motor symptoms, tremor, and cognitive dysfunctions) have been revealed.^14^ In addition, studies on the improvement of motor symptoms by noninvasive neuromodulatory stimulation in the motor symptom‐related areas in PD have also been reported^12^; however, for dysphagia in PD, symptom‐specific neuroanatomical substrates have not been identified, resulting in lacks of therapeutic trials with noninvasive stimulations.

Thus, to better understand the metabolic pathophysiology of dysphagia in PD and to investigate the therapeutic target of transcranial stimulation, we studied the altered distribution of brain glucose metabolism according to phase‐specific dysphagia in patients with PD. We hypothesized that there may be distributions of brain glucose metabolism specific to each phase of dysphagia.

## 2 METHODS

### 2.1 Participants

The study protocol was approved by the institutional review board of the Veterans Health Service Medical Center (2021‐11‐018), in accordance with the Declaration of Helsinki principles. As this was a retrospective cross‐sectional study, the need for informed consent was waived. Medical records of patients who met the following criteria were collected and reviewed from patients admitted to our hospital between January 2015 and December 2021. The inclusion criteria were as follows: (1) diagnosis of PD by a neurologist according to the UK Brain Bank Guidelines^15^; (2) underwent ^18^F‐FDG‐PET, T2‐weighted magnetic resonance imaging (MRI), and videofluoroscopic swallowing study (VFSS) at our hospital; and (3) Movement Disorder Society‐sponsored revision of the Unified Parkinson's Disease Rating Scale (UPDRS) scoring and Hoehn–Yahr (H–Y) staging performed within <1 month from VFSS.^16^

The exclusion criteria were as follows: (1) degenerative neurological disease other than PD such as progressive supranuclear palsy suspected on ^18^F‐FDG‐PET read by a nuclear medicine specialist; (2) more than 1 month between VFSS and ^18^F‐FDG‐PET; (3) UPDRS or H–Y scores not assessed within 1 month from VFSS; (4) comorbid conditions with central nervous system diseases other than PD; (5) evidence of cerebral lesions >3 mm in diameter on MRI^17^; (6) ventriculomegaly with Evans' index >0.4^18^; (7) cerebral white matter hyperintensities with Fazekas scale >2^19^; and (8) a history of major trauma or surgery in the neck.

For all patients, the following demographic and clinical information were collected: sex, age at the time of VFSS, more involved side of the body as judged by the UPDRS part III scores, chewing and swallowing scores of the UPDRS part II, H–Y stages, and period from the first diagnosis of PD to the date of VFSS.

### 2.2 Protocol of videofluoroscopic swallowing study

All VFSSs were performed using Logemann's protocol by the rehabilitation physician.^20^ To stabilize their anatomical position, participants were seated in an upright posture with their head position fixed, and lateral view radiographic images of the head, neck, and upper chest were taken in real time. Each patient was asked to swallow each of the following diets twice consecutively: 2 mL of water‐diluted barium (35% weight/volume), 2 mL of barium (35% weight/volume) mixed with curd yogurt, sliced bananas, boiled rice, and cookies (three cubes with dimensions of 1 cm). All radiographic videos were recorded as digital video files and analyzed by two rehabilitation medicine specialists until an agreement was reached.

The Videofluoroscopic Dysphagia Scale (VDS) was used as a scoring system which is a standardized and validated tool to rate 14 swallowing subitems (each of the oral and pharyngeal phases consists of seven subitems) that are scored during the VFSS.^21^ The seven subitems of oral phase consist of lip closure, bolus formation, mastication, swallowing apraxia, tongue‐to‐palate contact, premature bolus loss, and oral transit time. For pharyngeal phase, the seven subitems include vallecular residue, triggering of pharyngeal swallow, laryngeal elevation, pyriform sinus residue, coating of pharyngeal wall, pharyngeal transit time, and food aspiration into airways. Inter‐rater reliability of the VDS has been validated and this measure has been demonstrated to be statistically relevant with other etiologies as well.^21^, ^22^ The worst performance score across the 10 swallows (two swallows for each bolus and five types of bolus) for each bolus type was then determined as the overall impression score for each of the 14 subitems and used as the primary dependent variables.^7^ All recorded VFSSs were analyzed according to the VDS by two rehabilitation specialists who had more than 10 years of VFSS reading experience and were regular members of the Korean Dysphagia Society. When multiple VFSSs were performed, we analyzed the results for the date closest to the date of the PET scan.

The score for each subitem of the VFSS was assigned to binary and ordinal variables (the higher the score, the more severe the symptoms) based on the VDS. The scores for each subitem were input as the primary dependent variables, and the secondary dependent variables were oral and pharyngeal summative scores (zero to seven points for each phase).^23^

### 2.3 PET image acquisition

Routine ^18^F‐FDG‐PETs for screening to exclude other movement disorders were used for analyses. All patients fasted for at least 6 h and were verified to have a blood glucose level <180 mg/dL at the time of ^18^F‐FDG injection, and all antiparkinsonian medications were discontinued for at least 12 h prior to the scan. The participants were intravenously injected with a mean of 3.7 MBq/kg of ^18^F‐FDG, and PET images were obtained for 20 min using a Biograph 20 mCT PET/CT scanner (Siemens Medical Systems) 45 min after the injection. The time‐averaged PET scans were reconstructed with filtered back projection with a ramp filter producing images with a resolution of 3 mm full‐width at half‐maximum. The obtained raw PET image consisted of 400 × 400 × 110 voxels in three dimensions, and the size of each voxel was 1 × 1 × 1.5 mm^3^.

### 2.4 PET image preprocessing and analysis

Preprocessing was performed using Statistical Parametric Mapping 12 (Wellcome Trust Centre for Neuroimaging, https://www.fil.ion.ucl.ac.uk/spm/) and in‐house scripts with MATLAB 2020b (MathWorks Inc.; https://www.mathworks.com). First, reorientation was performed according to the anterior commissure of each raw PET image, and to consider minimal brain atrophy observed in patients, all PET scans from each patient were spatially normalized to the validated optimized ^18^F‐FDG‐PET template in the Montreal Neurological Institute (MNI)‐152 space (2 × 2 × 2 mm^3^ per voxel).^24^ All PET scans were then smoothened using an 8 mm full‐width at half‐maximum isotropic Gaussian kernel to improve the signal‐to‐noise ratio. The influence of global metabolism was removed by normalizing the count of each voxel to the total count of the cerebral voxels using proportional scaling. After preprocessing, all PET images were confirmed to be registered in the MNI‐152 space by a nuclear medicine specialist.

For voxel‐based analysis, each patient's preprocessed PET images were masked with the intracranial volume using Automated Anatomical Labeling atlas^3^, ^25^ and all the values of the voxels belonging to this volume were used as independent variables. Finally, of a total 592,895 (79 × 95 × 79) voxels in the smoothed whole‐brain volume, 170,998 voxels were included in each analysis. The neuroanatomical classifications of significant supratentorial regions were labeled using the Automated Anatomical Labeling atlas 3, and the infratentorial regions were labeled using the Spatially Unbiased Infra‐Tentorial atlas.^25^, ^26^ The coordinates of the voxels were presented according to the MNI‐152 space.

### 2.5 Statistical analysis

We tested all variables for normality using the Shapiro–Wilk test and visual data inspection with histograms; for ordinal dependent variables whose normality was rejected and all binary dependent variables, “normal” was coded as 0 and “impaired” as 1. Since the number of samples belonging to at least one group (case‐impaired or control‐intact) was <30 in most subitems and homogeneity of variance could not be assumed, the Wilcoxon rank‐sum test was used to compare the cases and controls for continuous and ordinal clinical variables. For the analyses of brain glucose metabolism for dysphagia, voxel‐wise regression analyses with dichotomized intactness for each VFSS subitem as the regressor of interest were implemented adjusting for age and PD duration at VFSS, which are valid negative predictors of swallowing.^27^, ^28^ Due to the variable case‐to‐control ratios in each of the 14 sub‐items of dysphagia, we reduced the bias of maximum likelihood estimate due to rare events (e.g., number of case or control <10) using two different analyses. First, we performed logistic regression with Firth's correction and profile penalized log likelihood, which is a robust technique that can be used to reduce rare event induced bias by assigning penalties.^29^, ^30^ Second, we performed multiple regression analyses using general linear model including small samples bias adjustment using adjusted residuals *e*_*i*_/(1 − *h*_*i*_)^1/2^ (where *h*_*i*_ is the diagonal element of the hat matrix *X*(*X*'*X*)^−1^ *X*′ corresponding to the observation of subject *i*) and nonparametric wild bootstrapping (9999 bootstraps).^31^, ^32^ To avoid interpretation complexity, we focused on Firth's correction, while results with the latter method, wild bootstrapping, were only described using phase‐specific overlapping strategy. For ordinal logistic regression model with the summative scores of oral and pharyngeal phases, respectively, as dependent variables, the assumption of proportionality was tested using the score test.^33^ In the case of a violated assumption, the thresholded and binarized metabolism maps from each subitem were overlapped to identify each region of hypo‐ or hypermetabolism related to each of the oral and pharyngeal phases of dysphagia. In case of subitem analyses, the significant regions were labeled using the peak beta values in the surviving clusters. For phase‐specific overlap analyses, the significant regions were labeled using the number of cluster overlaps.

A two‐tailed *p*‐threshold of 0.001 was applied to statistics for all voxel‐wise tests, uncorrected for multiple comparisons, because voxel‐wise corrections for multiple tests have been reported as being conservative especially when the region of interest was not restricted to a specific area.^34^, ^35^ For the cluster size‐based thresholding, clusters were considered significant when exceeded a cluster size threshold with *p* < 0.05 based on the global smoothness estimation on the whole‐brain volume computed with AFNI (version 22.1.14, https://afni.nimh.nih.gov/, 3dClustSim and 3dFWHMx),^36^, ^37^ and this yielded a three‐dimensional size threshold of *k* ≧ 91 contiguous voxels. Statistical analyses of the PET images and clinical variables were implemented using in‐house MATLAB scripts, SwE scripts in MATLAB,^32^ and the “logistf” package in R version 4.1.3 (R Foundation for Statistical Computing).

## 3 RESULTS

### 3.1 Participant characteristics

**FIGURE 1:**
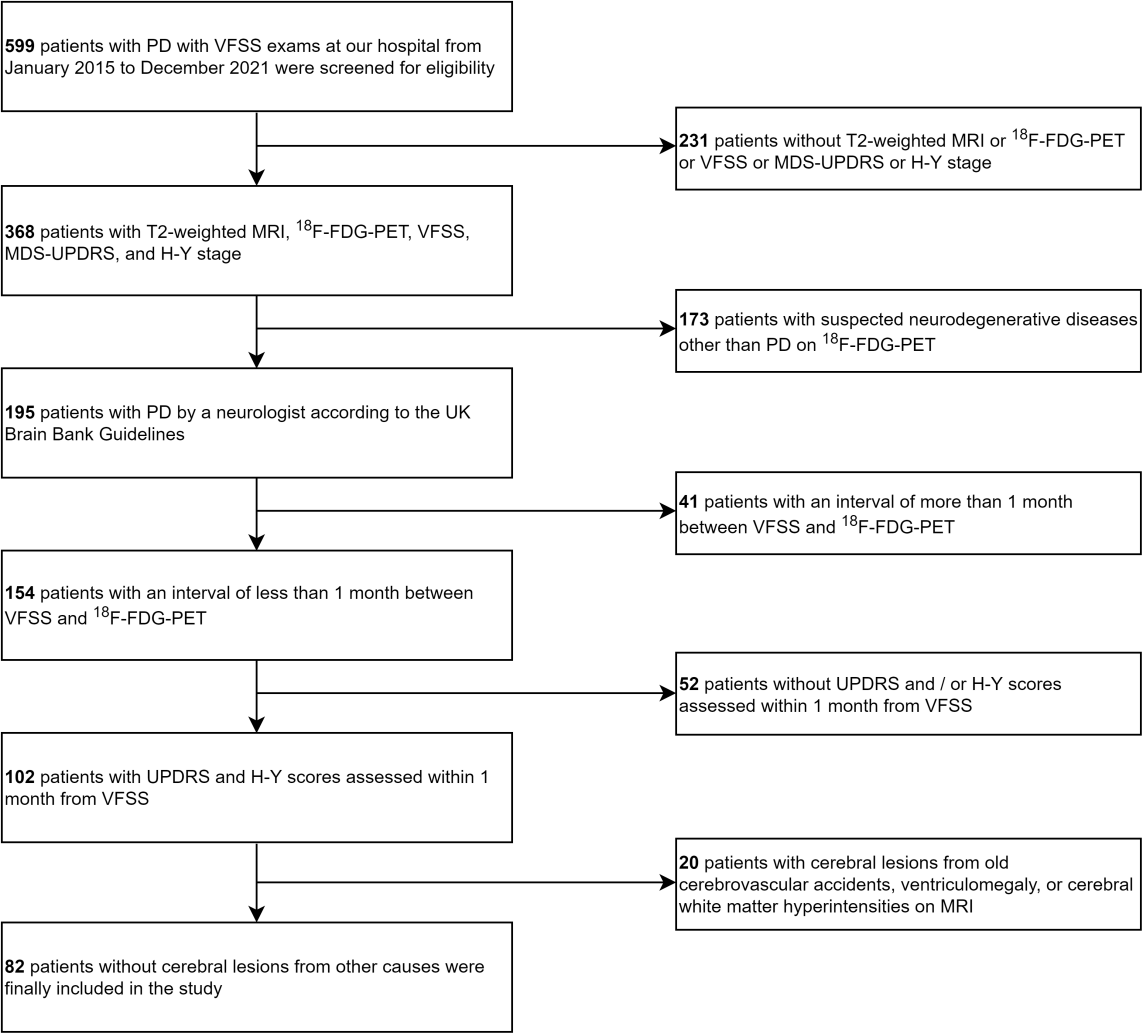
Flowchart of patient inclusion. ^18^F‐FDG‐PET, ^18^F‐fluoro‐2‐deoxy‐d‐glucose positron emission tomography; H–Y, Hoehn–Yahr; MRI, magnetic resonance imaging; PD, Parkinson's disease; UPDRS, Unified Parkinson's Disease Rating Scale; VFSS, videofluoroscopic swallowing study.

Figure 1 shows the inclusion flowchart. Among the 599 candidates diagnosed with PD, 82 patients who satisfied the inclusion criteria were finally included in the analysis. None of the included patients had any missing data for the variables of interest. The mean age ± standard deviation at the time of VFSS was 74.2 ± 5.8 years, and three patients (3.7%) were female (Table 1). The mean ± standard deviation of UPDRS total score was 71.2 ± 17.1 and the median (interquartile range) of H–Y stage was 4 (1). For each subitem, there was no statistically significant difference between cases and controls in age and PD duration at VFSS, while statistically significantly higher H–Y stage and UPDRS scores (part II‐chewing and swallowing, part III, and total scores) were found in the cases for several oral phase subitems (Table S1). Overlaid PET images after spatial and intensity normalization of all participants are shown in Figure S1.

**TABLE 1:** Demographic and clinical characteristics of the study population.

Parameters	Total (*n* = 82)
Age at VFSS, years	74.2 ± 5.8
Sex, No. (%)	
Male	79 (96.3)
Female	3 (3.7)
Clinical characteristics	
Parkinson's disease duration, years	13.5 ± 6.4
Hoehn–Yahr stage, median (IQR)	4 (1)
UPDRS part II chewing and swallowing score, median (IQR)	3 (1.5)
UPDRS part III score	45.3 ± 13.0
UPDRS total score	71.2 ± 17.1
Side of the body more involved, No. (%)^a^	
Right	39 (47.6)
Left	50 (41.0)
Equal	2 (2.4)

*Note*: For continuous variables, mean ± SD are shown.

Abbreviations: IQR, interquartile range; SD, standard deviation; UPDRS, Unified Parkinson's Disease Rating Scale; VFSS, videofluoroscopic swallowing study.

^a^ Judged by the UPDRS part III.

### 3.2 Dysphagia subitems and regional glucose metabolism using Firth's penalization

All subitem‐dependent variables in the VFSS were dichotomized as the normality of the distribution was rejected (Table 2; see Table S2 for distribution of dependent variable by subitem). For hypermetabolism, 10 (five for each of the oral and pharyngeal phases) out of 14 subitems as dependent variables represented at least one cluster composed of statistically significant voxels. And nine (four for the oral phase and five for the pharyngeal phase) subitems represented at least one cluster for hypometabolism.

**TABLE 2:** Case to control distribution by subitem score of dysphagia for voxel‐wise analyses.

Subitem^a^	Intact (control)	Impaired (case)
	*n* (%)	
Oral phase		
Lip closure	78 (95.1)	4 (4.9)
Bolus formation	74 (90.2)	8 (9.8)
Mastication	74 (90.2)	8 (9.8)
Swallowing apraxia	74 (90.2)	8 (9.8)
Tongue‐to‐palate contact	78 (95.1)	4 (4.9)
Premature bolus loss	42 (51.2)	40 (48.8)
Oral transit time	55 (67.1)	27 (32.9)
Pharyngeal phase		
Vallecular residue	19 (23.2)	63 (76.8)
Triggering of pharyngeal swallow	44 (53.7)	38 (46.3)
Laryngeal elevation	22 (26.8)	60 (73.2)
Pyriform sinus residue	34 (41.5)	48 (58.5)
Coating of pharyngeal wall	53 (64.6)	29 (35.4)
Pharyngeal transit time	74 (90.2)	8 (9.8)
Food aspiration into airways	17 (20.7)	65 (79.3)

^a^ Assessed based on the Videofluoroscopic Dysphagia Scale.

Impaired lip closure, the first significant subitem of the oral phase, was associated with bilateral inferior temporal gyrus hypermetabolism (Figure S2A). Second, impaired bolus formation was associated with hypometabolism in the left cerebellar lobule X (Figure S2B). Third, impaired mastication was associated with hypermetabolism of the bilateral anterior cingulate and orbital parts of middle frontal gyri, left precuneus (Figure S2C). In addition, hypometabolism of the bilateral superior temporal poles and orbital parts of inferior frontal gyrus was also related to the impaired mastication. Fourth, presence of swallowing apraxia was associated with hypermetabolism of the right cerebellar lobule III, crus I and II, and left cerebellar lobule III, VI, and crus I (Figure S2D). Hypometabolism of the superior temporal gyrus was also associated with the presence of swallowing apraxia. Fifth, impaired tongue‐to‐palate contact was associated with hypermetabolism of the right inferior temporal gyrus (Figure S2E). Sixth, there was no significant cluster associated with the presence of premature bolus loss. Seventh, delayed oral transit time was associated with hypermetabolism of the right middle temporal pole, left fusiform gyrus, cerebellar lobule V and VIII, and bilateral rectus and anterior cingulate cortices (Figure S2F). Hypometabolism of the bilateral orbital parts of inferior to middle frontal and right angular gyri, and precuneus was related to the delayed oral transit time.

Presence of vallecular residue, the first significant subitem of the pharyngeal phase, was associated with hypometabolism of the bilateral medial aspects of superior frontal gyrus (Figure S3A). Second, impaired triggering of pharyngeal swallow was associated with hypermetabolism of the right cerebellar lobule VI (Figure S3B). Hypometabolism of the middle frontal gyrus was also associated with impaired triggering. Third, there was no significant cluster associated with the impaired laryngeal elevation. Fourth, presence of pyriform sinus residue was associated with hypermetabolism of the right inferior temporal gyrus (Figure S3C). Additionally, hypometabolism of the triangular part of left inferior frontal and medial aspects of superior frontal gyri was also related to the presence of residue. Fifth, presence of coating of pharyngeal wall was associated with hypermetabolism of the right cerebellar crus II, lobule VIII, superior occipital gyrus, and left cerebellar lobule V (Figure S3D). Hypometabolism of the bilateral insula, triangular part of right inferior frontal gyrus, and medial aspects of left superior frontal gyrus was also related to the presence of coating of pharyngeal wall. Sixth, delayed pharyngeal transit time was associated with hypermetabolism of the bilateral cerebellar lobule III (Figure S3E). Seventh, presence of food aspiration into airways was associated with hypermetabolism of the left superior occipital gyrus (Figure S3F). Hypometabolism of the bilateral middle to superior frontal and anterior cingulate cortices and superior temporal poles was related to the presence of food aspiration.

### 3.3 Phase‐specific dysphagia and regional glucose metabolism

The voxel‐wise proportional odds model for summative scores of oral and pharyngeal phases violated the assumption of proportionality, so the subitem clusters that survived the thresholds in oral and pharyngeal phase were binarized and overlapped for each phase (Table 3).

**TABLE 3:** Regions with at least two overlaps of significant clusters.

Cluster	Maximum number of overlaps	Volume (voxels)	Composition^a^ (%)
*Oral phase: hypermetabolism*			
1	3	737	R inferior temporal gyrus (54.3) R temporal pole (middle temporal gyrus) (34.6)
2	2	463	R anterior cingulate cortex, pregenual (43.6)
3	2	392	L anterior cingulate cortex, pregenual (32.1) L superior frontal gyrus, medial orbital (29.1)
*Oral phase: hypometabolism*			
1	2	274	L inferior frontal gyrus, orbital part (63.5) L inferior frontal gyrus, triangular part (25.5)
*Pharyngeal phase: hypermetabolism*			
None			
*Pharyngeal phase: hypometabolism*			
1	2	3424	L superior frontal gyrus, dorsolateral (20.6)
2	2	976	R superior frontal gyrus, dorsolateral (52.8) R middle frontal gyrus (29.1)
3	2	618	R insula (25.9) R posterior orbital gyrus (22.8) R temporal pole (superior temporal gyrus) (22.8)

Abbreviations: L, left; R, right.

^a^ Only structures occupying more than 20% of each cluster are presented.

**FIGURE 2:**
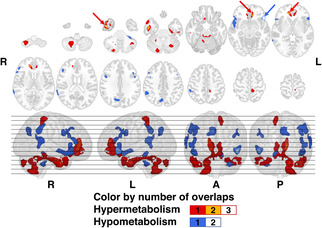
Overlay maps showing association of brain glucose metabolism with the oral phase‐specific dysphagia. Red and blue arrows respectively point to regions of increased or decreased metabolism that showed at least two overlaps of significant clusters. Integers in the color bar indicates the phase‐specific numbers of overlaps of the clusters surviving the threshold (two‐tailed voxel‐wise *p*‐threshold of 0.001, cluster volume of at least 91 voxels) in the Firth's penalized binary logistic regression for each subitem controlling the age and Parkinson's disease duration at the time of the VFSS. The MNI‐152 *z*‐coordinates of each row of the slices were as follows: −60 −50 −40 −30 −20 −10 0; 10 20 30 40 50 60 70 in ventro‐dorsal orders, respectively. A, anterior; L, left; MNI, Montreal Neurological Institute; P, posterior; R, right; VFSS, videofluoroscopic swallowing study.

The regions with the highest overlap number of oral phase‐specific hypermetabolic clusters were concentrated in the right inferior temporal and lateral aspects bilateral anterior cingulate cortices (Figure 2). On the other hand, the hypometabolic region showing the highest number of overlaps was over the left orbital parts of inferior to middle frontal gyri.

**FIGURE 3:**
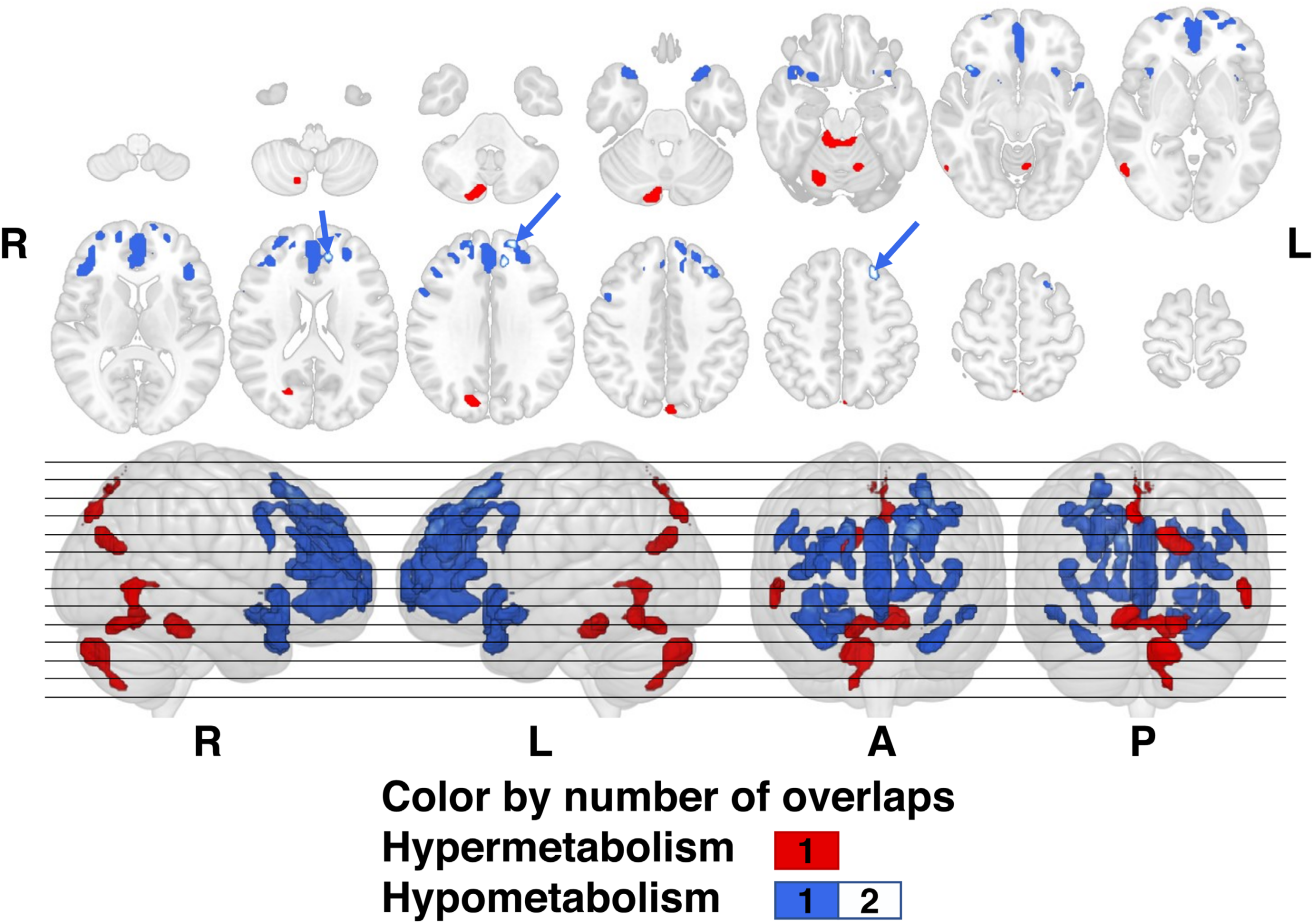
Overlay maps showing association of brain glucose metabolism with the pharyngeal phase‐specific dysphagia. Blue arrows point to regions of hypometabolism that showed two overlaps of significant clusters. Integers in the color bar indicates the phase‐specific numbers of overlaps of the clusters surviving the threshold (two‐tailed voxel‐wise *p*‐threshold of 0.001, cluster volume of at least 91 voxels) in the Firth's penalized binary logistic regression for each subitem controlling the age and Parkinson's disease duration at the time of the VFSS. The MNI‐152 *z*‐coordinates of each row of the slices were as follows: −60 −50 −40 −30 −20 −10 0; 10 20 30 40 50 60 70 in ventro‐dorsal orders, respectively. A, anterior; L, left; MNI, Montreal Neurological Institute; P, posterior; R, right; VFSS, videofluoroscopic swallowing study.

Although hypermetabolism associated with the pharyngeal phase dysfunction was distributed over the posterior aspects of the bilateral parietal lobes and cerebellum, the maximum number of overlaps was only one (Figure 3). However, for hypometabolic distribution associated with the pharyngeal phase‐specific dysphagia, the overlap map generated showed the highest number of overlaps in the medial aspects of anterior cingulate and middle to superior frontal gyri.

The dysphagia phase‐specific topologic distributions obtained using adjusted residuals and wild bootstrapping were similar to the results using Firth's penalization, although the overall volume of significant clusters was reduced in the results from the former method (Figures S4 and S5).

## 4 DISCUSSION

We hereby present a neurophysiological basis for understanding the PD‐related dysphagia. Although there were differences in the significant metabolic regional distribution for each subitem even in the same phase, the neuroanatomical areas related to the impaired swallowing‐related movement at multiple phases were identified by overlapping surviving clusters. In the case of oral phase dysphagia, hypermetabolism of the bilateral (right > left) cerebellum and ventral aspect of the temporal lobe, and medial frontal lobe were mainly associated with the dysfunction. Decreased metabolism of the bilateral temporal poles and orbital parts of frontal lobe was also commonly associated with the oral phase swallowing impairments (Figure 2). For the dysphagia of pharyngeal phase, hypermetabolism of posterior parts of the parietooccipital lobe, cerebellum, and hypometabolism of the anterior parts of the frontotemporal lobe were found to be related to the dysfunctions (Figure 3).

Regional hypometabolism in PD has been suggested to represent areas of dysfunction that are directly or indirectly related to parkinsonian symptoms.^38^ For the bilateral temporal poles, which showed hypometabolism associated with oral phase dysphagia in our analyses, a previous study revealed that decreased cortical volume and blood oxygen level‐dependent (BOLD) signals of this area were associated with early Alzheimer's disease‐related dysphagia.^39^ Additionally, this region has been found to be essential to human swallowing through studies using PET and functional MRI (fMRI).^40^, ^41^ In a previous study using fMRI in patients with PD and another study using lesion symptom mapping in patients with stroke, the temporal poles and orbital parts of frontal lobe, which were shown to be associated with the dysphagia of oral phase in our results, were also found to be significantly correlated to the occurrence of dysphagia.^42^, ^43^ Although the distribution was more ventral than that in the oral phase, it was found that the hypometabolism in the temporal poles was also associated with the presence of food aspiration into airways (Figure S3F).

In a previous study using ^18^F‐FDG‐PET in PD, hypometabolism of the anterior cingulate and medial parts of middle to superior frontal gyri, which were shown to be associated with pharyngeal phase dysphagia in our study, have also been reported to be associated with delayed initiation of pharyngeal swallowing.^44^ Additionally, these regions are reported to be related to the normal swallowing process in humans.^6^

In previous studies using scaled subprofile model and principal component analysis, distribution of brain glucose metabolism in PD has been characterized by hypermetabolism of putamen, pallidum, thalamus, sensorimotor cortex, pons and cerebellum, and hypometabolism of the posterior temporoparietal, occipital, and frontal lobes.^45^, ^46^ Among the metabolic distributions, cerebellar hypermetabolism was found in all PD‐related brain glucose metabolism studies, consistent with the metabolic distribution related to dysphagia in our study, although the analytical methods were different. This regional metabolic increase in patients with PD may reflect both maladaptive and compensatory effects.^47^ In terms of homunculi obtained via task‐specific fMRI studies, regions coordinates the movements of lips and mouth are located bilaterally in the cerebellar lobules VI to VIII,^48^ which is in line with our results.

In our study, increased metabolism of ventral aspect of the right inferior temporal lobe was also significantly associated with oral phase dysphagia and presence of pyriform sinus residue. This region shows a significant increase in regional cerebral blood flow in human voluntary swallowing,^49^ and in a study using fMRI, this area showed overactivity in patients with functional dysphagia; however, the symptoms were resolved with treatment by transcranial magnetic stimulation, and at the same time, the BOLD signal was normalized.^13^ The dysphagia associated with the BOLD overactivity of the right inferior temporal lobe is consistent with our findings for dysphagia associated with regional hypermetabolism.

Increased metabolism of the right cerebellar crus II and bilateral cerebellar lobule III was observed in both the oral and pharyngeal phases of dysphagia (Figures 2 and 3). In a previous experimental study using cerebellar electrodes in rodents, cerebellar crus II showed roles of central pattern generating and fine coordination of licking behavior.^50^ However, since studies on the role of the crus II or lobule III in human swallowing process have not been reported, it cannot be determined whether this finding in our study has a direct causal relationship with dysphagia or is due to the cerebellar pathophysiology of advanced PD.

Hypermetabolism of ventrolateral aspect of the anterior cingulate cortex showed association with the development of oral phase dysphagia. However, hypometabolism in the dorsomedial aspect of the anterior cingulate cortex was related to development of dysphagia of pharyngeal phase. Although it has been commonly reported that the anterior cingulate cortex is recruited during human swallowing in a number of studies, we report for the first time that an increase or decrease in cerebral glucose metabolism, depending on the relative location, is associated with each phase of dysphagia.

Our presentation has several limitations. First, since the institution where the study was conducted was a hospital for veterans, the demographic characteristics of patients visiting the hospital were biased toward male sex, making it difficult to consider sex‐related factors. Second, the statistical power of some dependent variables may have decreased because the case–control ratios were different for each subitem‐dependent variable. We used two different methods (Firth's penalization/adjusted residuals with wild bootstrapping) to reduce the bias of maximum likelihood estimates due to rare events; nevertheless, statistically significant neuroanatomical regions may not have been detected due to the unbalanced case–control ratio per group. Third, administration of antiparkinsonian medications was discontinued at least 12 h prior to the ^18^F‐FDG‐PET scans, but for VFSS, it was not possible to confirm whether each patient was in the on‐ or off‐state at the time of the evaluation, so it could not be entered as a nuisance variable. Fourth, we tried to perform the ordinal logistic regression using the summative scores of each phase, but the proportional odds assumption was violated, leading to the suboptimal choice of the overlapping methodology.

## 5 CONCLUSIONS

This study provides unprecedented insights into the relationship between each phase of dysphagia and altered brain glucose metabolism in PD. To enable clinicians to use our results to identify patients with PD at risk for phase‐specific dysphagia, our report requires further corroboration using longitudinal iterative analyses of metabolic changes during the progression of dysphagia over time in patients with PD. In addition, it is necessary to confirm whether phase‐specific dysphagia is alleviated through neuromodulatory transcranial stimulation of the corresponding cortical regions.

## AUTHOR CONTRIBUTIONS

**Ji Yeon Oh**: Data curation, investigation, resources, visualization, and writing—original draft preparation. **Eui Jin An**: Data curation, investigation, and writing—review and editing. **Young Lee**: Formal analysis, software, validation, and writing—review and editing. **Seung Min Kim**: Conceptualization, methodology, resources, supervision, and writing—review and editing. **Miju Cheon**: Formal Analysis, methodology, software, validation, and writing—review and editing. **Jun Yup Kim**: Conceptualization, data curation, formal analysis, funding acquisition, project administration, resources, software, supervision, writing—original draft preparation, and writing—review and editing.

## FUNDING INFORMATION

This study was partly supported by a VHS Medical Center Research Grant, South Korea (VHSMC20048). The views expressed are those of the authors and not necessarily those of the funder.

## CONFLICT OF INTEREST STATEMENT

The authors declared no potential conflicts of interest with respect to the research, authorship, and/or publication of this article.

## PATIENT CONSENT STATEMENT

Not applicable due to the retrospective nature of this study.

## Supporting information


Data S1.

## Data Availability

The de‐identified data and codes that support the findings of this study are available from the corresponding author upon reasonable request.
