# Supplementary material for: Association of dysphagia with altered brain glucose metabolism in Parkinson's disease
Source: CNS Neurosci Ther. 2023 Apr 11;29(9):2498–507. doi: 10.1111/cns.14214 (PMC10401099; doi:10.1111/cns.14214)
Supplement: Supplementary file 1 — Data S1. [file CNS-29-2498-s001.pdf]

Supplementary Table 1. Parkinson's disease duration and symptom severity by impairment for each sub-item according to the Videofluoroscopic Dysphagia Scale

Sub-item	Impairment	*n* (%)	Age at VFSS, years		Parkinson's disease duration, years		Hoehn-Yahr stage		UPDRS part II-chewing and swallowing		UPDRS part III		UPDRS total	
			Median (IQR)	*p*	Median (IQR)	*p*	Median (IQR)	*p*	Median (IQR)	*p*	Median (IQR)	*p*	Median (IQR)	*p*
**Oral phase**														
Lip closure	Intact	78 (95)	74 (6)	0.50	13 (12)	0.71	4 (1)	0.03*	3 (2)	0.05*	44.5 (20)	< 0.01*	71.5 (29)	0.01
	Impaired	4 (5)	76.5 (14)		14 (7.5)		4.5 (1)		4 (0.5)		65.5 (10)		99 (11)	
Bolus formation	Intact	74 (90)	73.5 (5)	0.10	12.5 (12)	0.15	4 (1)	0.01*	3 (1)	< 0.01*	43 (19)	< 0.01*	69 (29)	< 0.01*
	Impaired	8 (10)	78.5 (11)		16.5 (7)		4.5 (1)		4 (0)		66 (9.5)		98.5 (6.5)	
Mastication	Intact	74 (90)	74 (6)	0.81	13 (10)	0.10	4 (1)	0.06	3 (1)	< 0.01*	43 (19)	< 0.01*	69 (30)	< 0.01*
	Impaired	8 (10)	73 (11.5)		18.5 (7.5)		4 (0.5)		4 (0.5)		60.5 (9)		92.5 (15)	
Swallowing apraxia	None	74 (90)	73.5 (5)	0.20	13 (12)	0.72	4 (1)	0.27	3 (1)	< 0.01*	43 (19)	< 0.01*	69 (29)	< 0.01*
	Present	8 (10)	75.5 (12.5)		12 (13.5)		4 (2)		4 (0.5)		58.5 (14)		89.5 (10)	
Tongue-to-palate contact	Intact	78 (95)	74 (6)	0.75	13 (12)	0.44	4 (1)	0.09	3 (2)	0.18	44.5 (20)	0.01	71.5 (29)	0.04
	Impaired	4 (5)	71.5 (6.5)		14 (5.5)		4 (0.5)		3.5 (1)		60.5 (12)		89 (20)	
Premature bolus loss	None	42 (51)	73.5 (10)	0.97	12 (10)	0.16	4 (1)	0.23	3 (1)	< 0.01*	34.5 (20)	< 0.01*	56 (29)	< 0.01*
	Present	40 (49)	74 (4)		13.5 (11)		4 (1)		3 (1)		50 (13)		81.5 (18)	
Oral transit time	Intact	55 (67)	73 (5)	0.35	13 (11.5)	0.39	4 (1)	0.07	3 (1)	< 0.01*	40 (14.75)	< 0.01*	62 (27)	< 0.01*
	Delayed	27 (33)	74 (7)		14 (10.75)		4 (1.75)		4 (1)		58 (11)		88 (16.75)	

**Pharyngeal phase**														
Vallecular residue	None	19 (23)	73.5 (6)	0.74	13 (12)	0.87	4 (1)	0.17	3 (1.5)	0.49	43.5 (17)	0.97	69 (27.5)	0.35
	Present	63 (77)	74 (6)		13 (9)		3 (1)		3 (2)		47 (23)		79 (29)	
Triggering of pharyngeal swallow	Intact	44 (54)	73 (8.25)	0.73	13 (6.75)	0.69	3 (1)	0.45	3 (1.75)	0.46	49 (17.5)	0.05	78 (23.5)	0.20
	Impaired	38 (46)	74 (5.75)		13 (12)		4 (1)		3 (2)		43 (20)		70 (29.75)	
Laryngeal elevation	Intact	22 (27)	74 (5)	0.21	10.5 (13)	0.31	3.5 (1)	0.28	3 (1)	0.47	45.5 (13)	0.29	74 (21)	0.66
	Impaired	60 (73)	72.5 (6.5)		13 (10)		4 (1)		3 (2)		44.5 (21.5)		72.5 (32)	
Pyriform sinus residue	None	34 (41)	72.5 (9)	0.56	13 (10)	0.93	4 (1)	0.90	3 (2)	0.87	47.5 (19)	0.90	74 (33)	0.61
	Present	48 (59)	74 (5)		13 (12)		4 (1)		3 (2)		43.5 (20.5)		74 (28)	
Coating of pharyngeal wall	None	53 (65)	74 (8.5)	0.38	13 (11.25)	0.74	4 (1)	0.40	3 (1)	0.11	45 (19.25)	0.91	75 (32.75)	0.37
	Present	29 (35)	73 (4.25)		13 (11.25)		4 (1)		3 (1.25)		43 (20.75)		73 (24.5)	
Pharyngeal transit time	Intact	74 (90)	73.5 (6)	0.49	13 (12)	0.88	4 (1)	0.09	3 (2)	0.14	45 (19)	0.24	73 (30)	0.08
	Delayed	8 (10)	75 (6.5)		13 (11.5)		4 (1.5)		3.5 (1)		53.5 (24.5)		86 (31)	
Food aspiration into airways	None	17 (21)	71 (6.25)	0.12	12 (5.75)	0.55	4 (1)	0.68	3 (2)	0.53	35 (17.25)	0.13	63 (27)	0.07
	Present	65 (79)	74 (5.5)		13 (12)		4 (1)		3 (2)		46 (16.75)		75 (29)	

Abbreviations: IQR, interquartile range; UPDRS, Unified Parkinson's Disease Rating Scale; VFSS, videofluoroscopic swallowing study.

*Statistically significant at two-tailed threshold of $p < 0.05$.

Supplementary Table 2. Videofluoroscopic Dysphagia Scale sub-item scores for voxel-wise analyses

Sub-item	Coded as (dichotomized)			Coded as (raw)		
	**Meaning**	**Scores**	***n* (%)**	**Meaning**	**Scores**	***n* (%)**
**Oral phase**						
Lip closure	Intact	1	78 (95)	Intact	0	78 (95)
	Impaired	0	4 (5)	Inadequate	1	4 (5)
				None	2	0 (0)
Bolus formation	Intact	1	74 (90)	Intact	0	74 (90)
	Impaired	0	8 (10)	Inadequate	1	6 (7)
				None	2	2 (2)
Mastication	Intact	1	74 (90)	Intact	0	74 (90)
	Impaired	0	8 (10)	Inadequate	1	6 (7)
				None	2	2 (2)
Swallowing apraxia	None	1	74 (90)	None	0	74 (90)
	Present	0	8 (10)	Mild	1	5 (6)
				Moderate	2	2 (2)
				Severe	3	1 (1)
Tongue-to-palate contact	Intact	1	78 (95)	Intact	0	78 (95)
	Impaired	0	4 (5)	Inadequate	1	3 (4)
				None	2	1 (1)
Premature bolus loss	None	1	42 (51)	None	0	42 (51)
	Present	0	40 (49)	<10%	1	10 (12)
				10–50%	2	13 (16)
				>50%	3	17 (21)
Oral transit time	Intact	1	55 (67)	≤1.5 s	0	55 (67)
	Delayed	0	27 (33)	>1.5 s	1	27 (33)
**Pharyngeal phase**						
Vallecular residue	None	1	19 (23)	None	0	19 (23)
	Present	0	63	<10%	1	17

			(77)			(21)
				10–50%	2	23 (28)
				>50%	3	23 (28)
Triggering of pharyngeal swallow	Intact	1	44 (54)	Normal	0	44 (54)
	Impaired	0	38 (46)	Delayed	1	38 (46)
Laryngeal elevation	Intact	1	22 (27)	Normal	0	22 (27)
	Impaired	0	60 (73)	Impaired	1	60 (73)
Pyriform sinus residue	None	1	34 (41)	None	0	34 (41)
	Present	0	48 (59)	<10%	1	17 (21)
				10 – 50%	2	19 (23)
				>50%	3	12 (15)
Coating of pharyngeal wall	None	1	53 (65)	No	0	53 (65)
	Present	0	29 (35)	Yes	1	29 (35)
Pharyngeal transit time	Intact	1	74 (90)	≤1.0 s	0	74 (90)
	Delayed	0	8 (10)	>1.0 s	1	8 (10)
Food aspiration into airways	None	1	17 (21)	None	0	17 (21)
	Present	0	65 (79)	Supraglottic penetration	1	16 (20)
				Subglottic aspiration	2	49 (60)

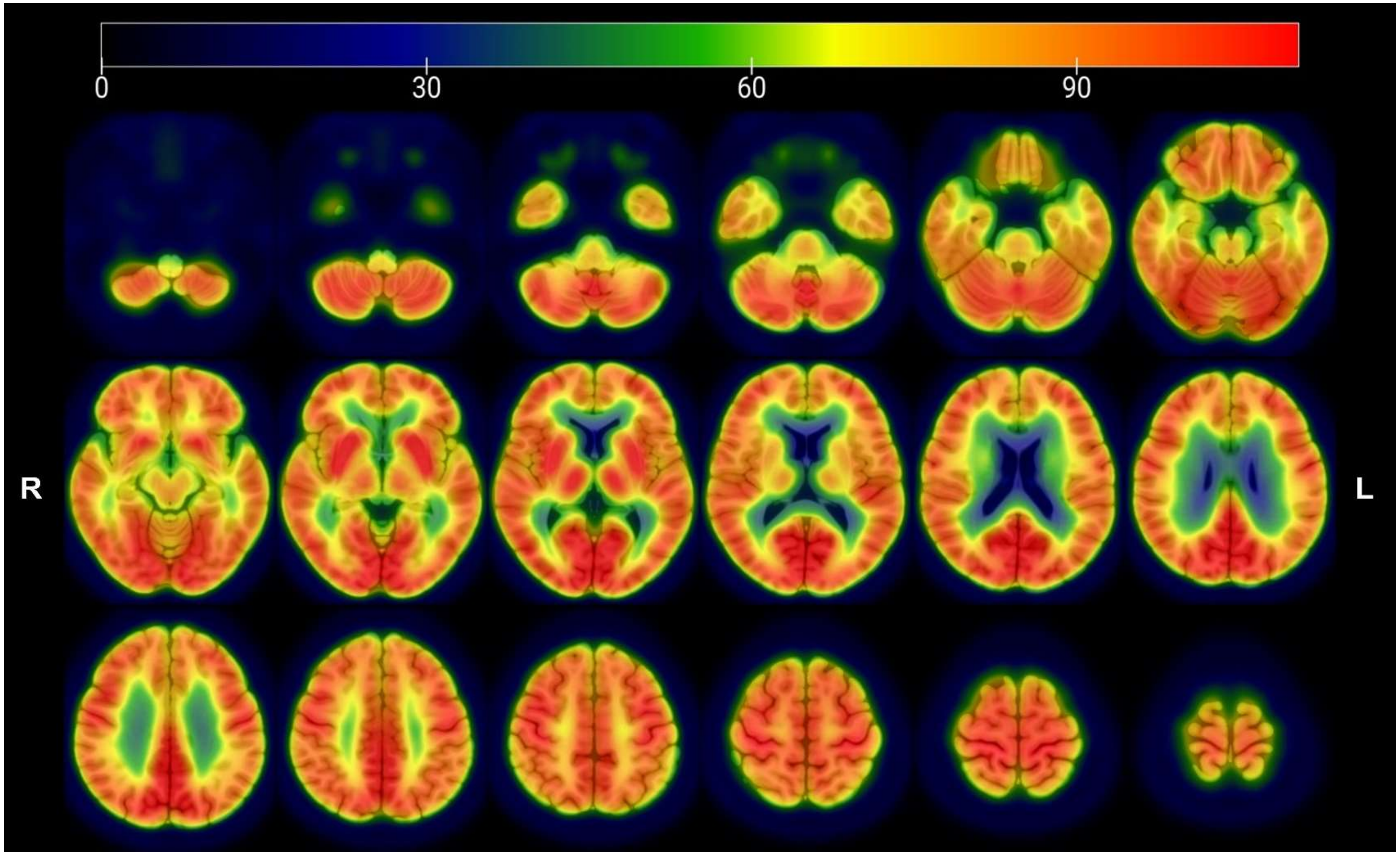

Supplementary Figure 1. Overlay maps of spatial and intensity normalized ^{18}F-FDG PET images from all study subjects. The color bar shows the sum of the intensity-normalized uptake values. The MNI-152 z-coordinates of each row of the slices were -60 -52 -44 -36 -28 -20; -12 -4 4 12 20 28; and 36 44 52 60 68 76 in ventro-dorsal orders, respectively. ^{18}F-FDG PET, ^{18}F-fluorodeoxyglucose positron emission tomography; MNI, Montreal Neurological Institute; L, left; R, right.

(A) Impaired lip closure

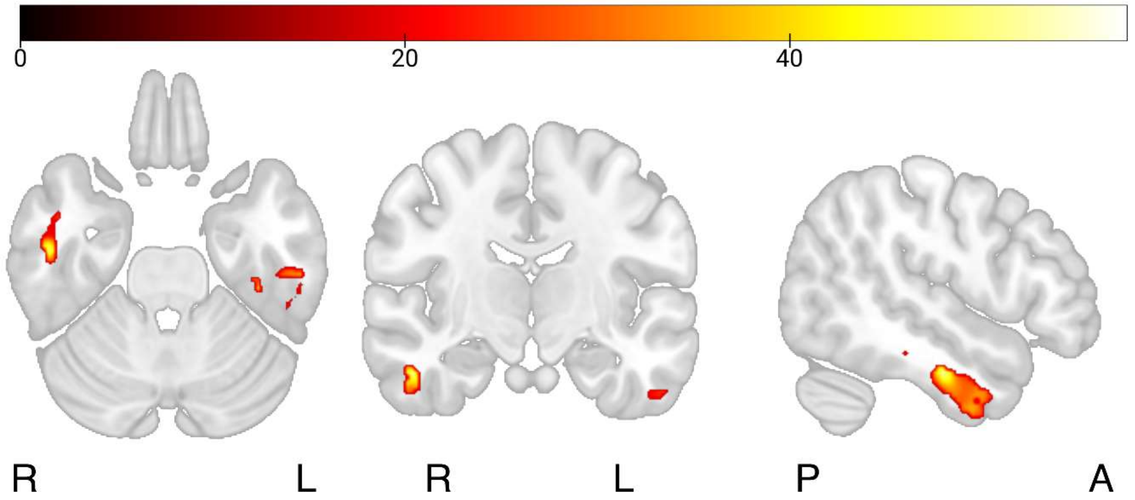

(B) Impaired bolus formation

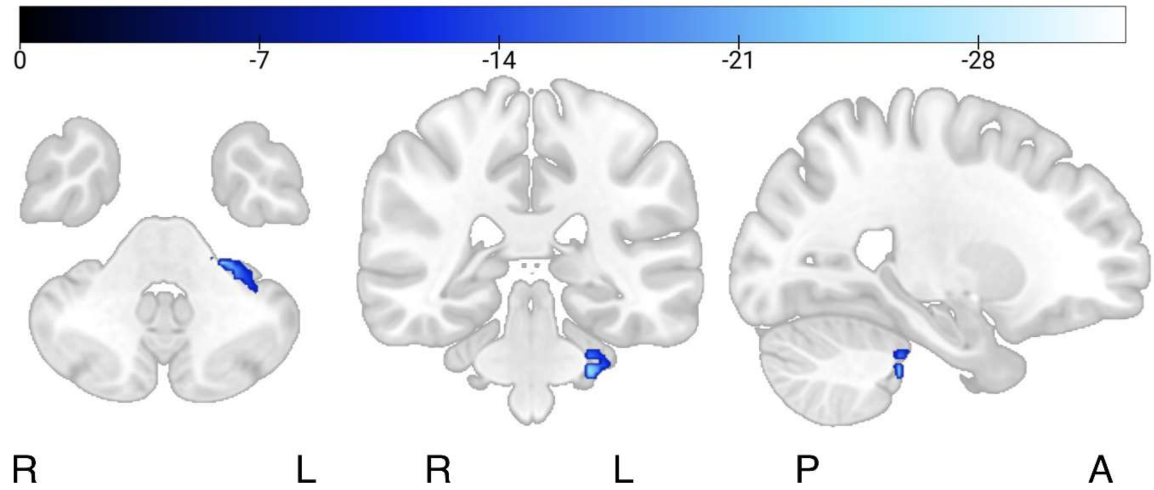

(C) Impaired mastication

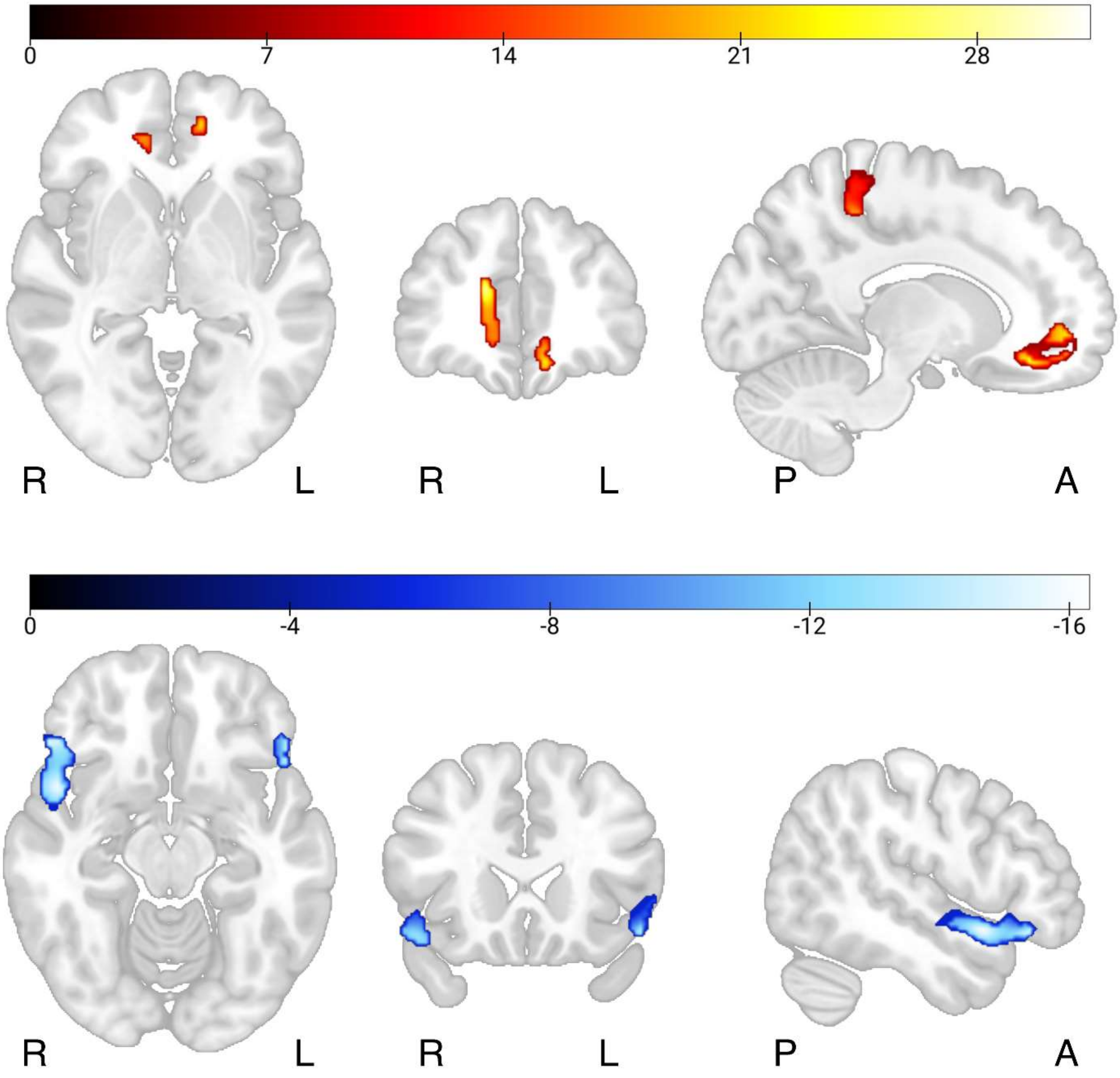

(D) Presence of swallowing apraxia

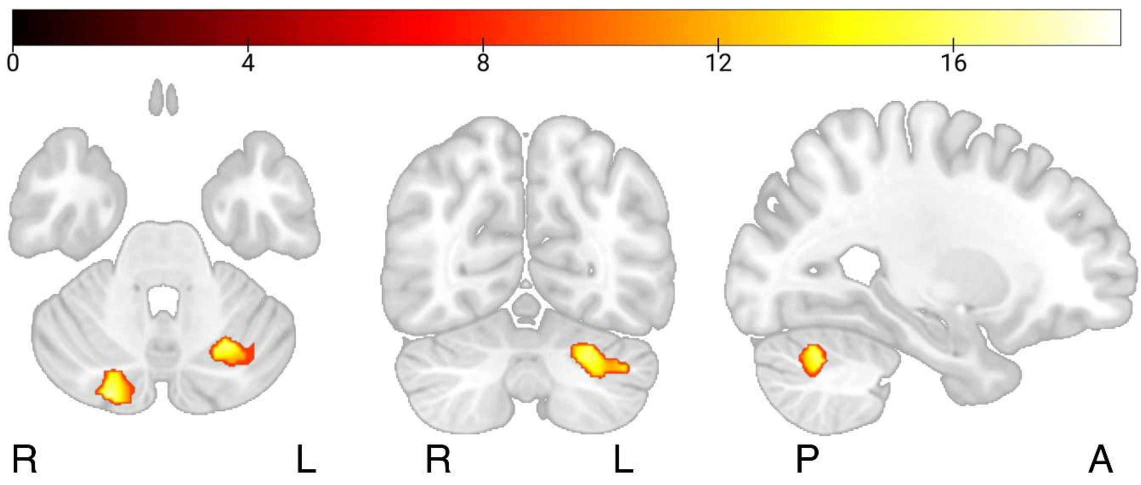

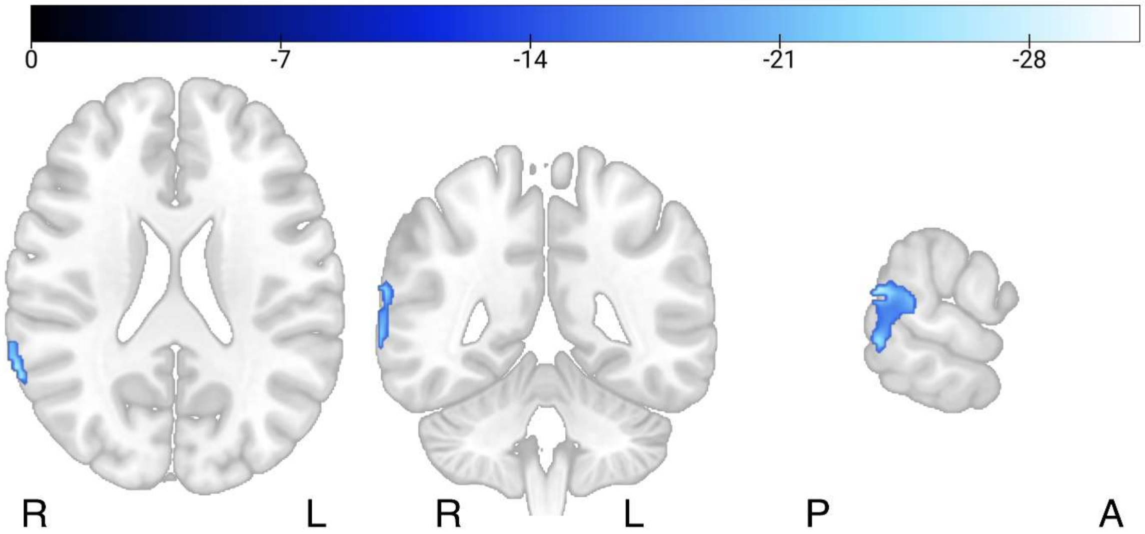

(E) Impaired tongue-to-palate contact

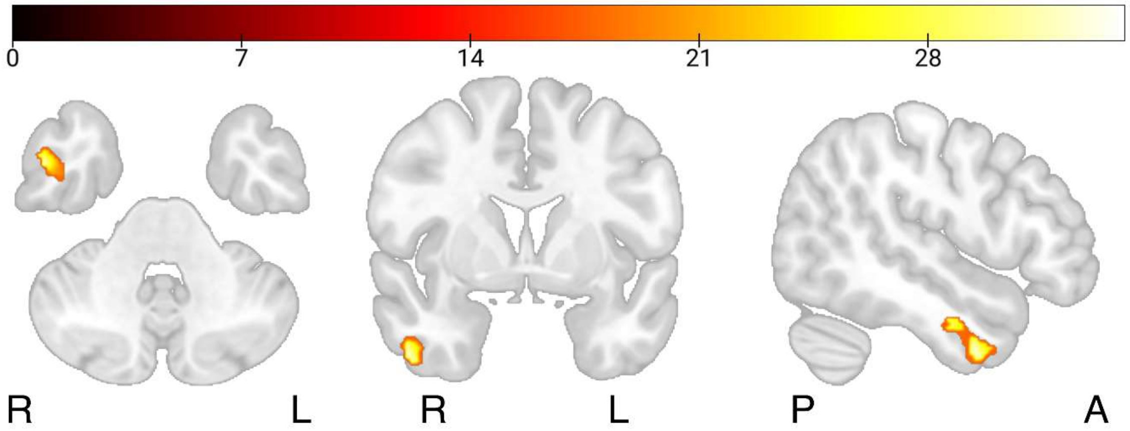

(F) Delayed oral transit time

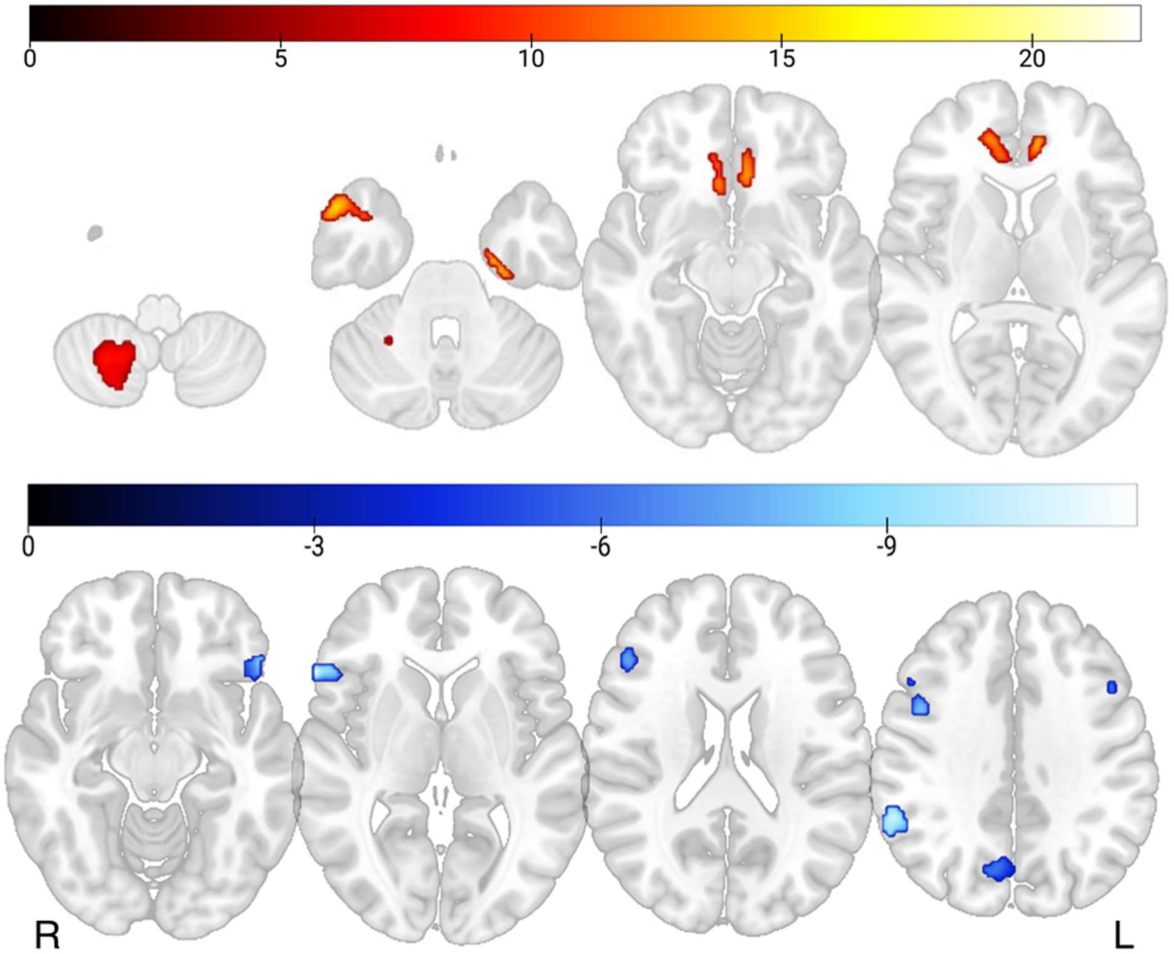

Supplementary Figure 2. Statistical maps showing the metabolic distribution of glucose associated with each dysphagia sub-item in the oral phase. (**A**) impaired lip closure, (**B**) impaired bolus formation, (**C**) impaired mastication, (**D**) presence of swallowing apraxia, (**E**) impaired tongue-to-palate contact, and (**F**) delayed oral transit time. Only voxels contained in the surviving clusters are displayed. Beta values from the Firth's penalized binary logistic regression were calculated using normalized voxel-wise uptake values and each of the sub-item scores controlling the age and Parkinson's disease duration at the time of the VFSS. The color bar shows beta values: a positive number indicates hypermetabolism, and a negative number indicates hypometabolism. A, anterior; L, left; MNI, Montreal Neurological Institute; P, posterior; R, right; VFSS, videofluoroscopic swallowing study.

(A) Presence of vallecular residue

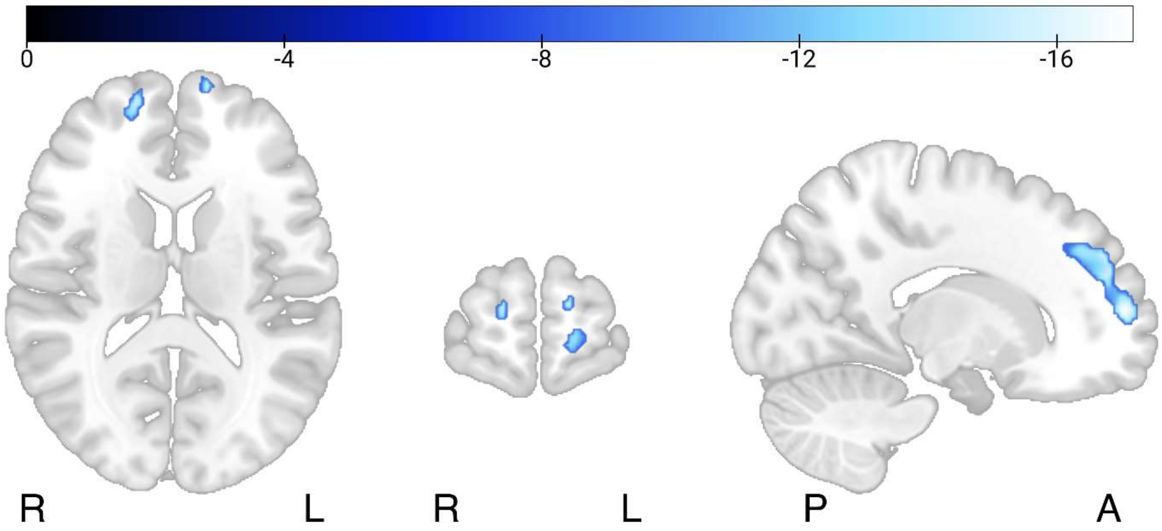

(B) Impaired triggering of pharyngeal swallow

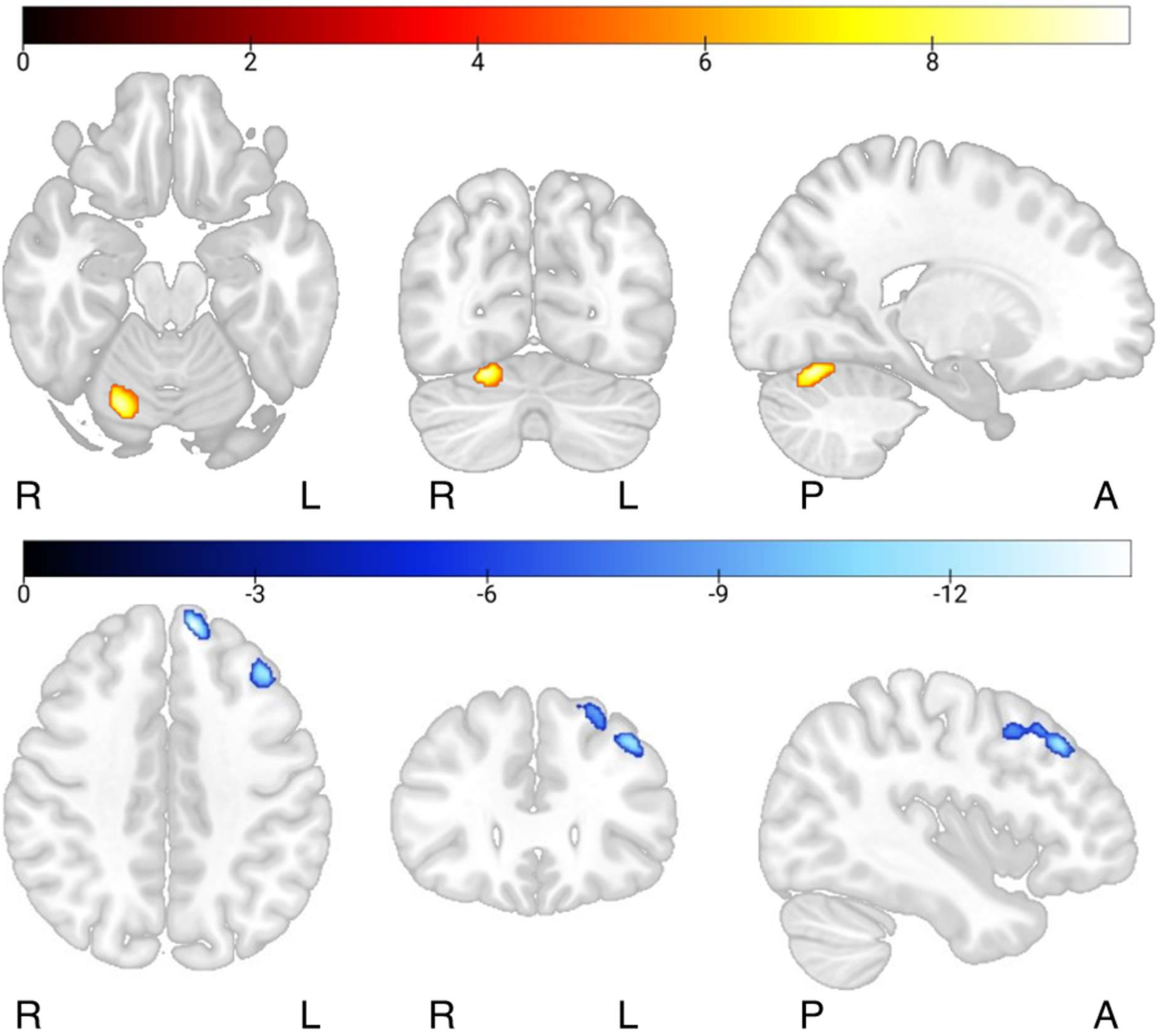

(C) Presence of pyriform sinus residue

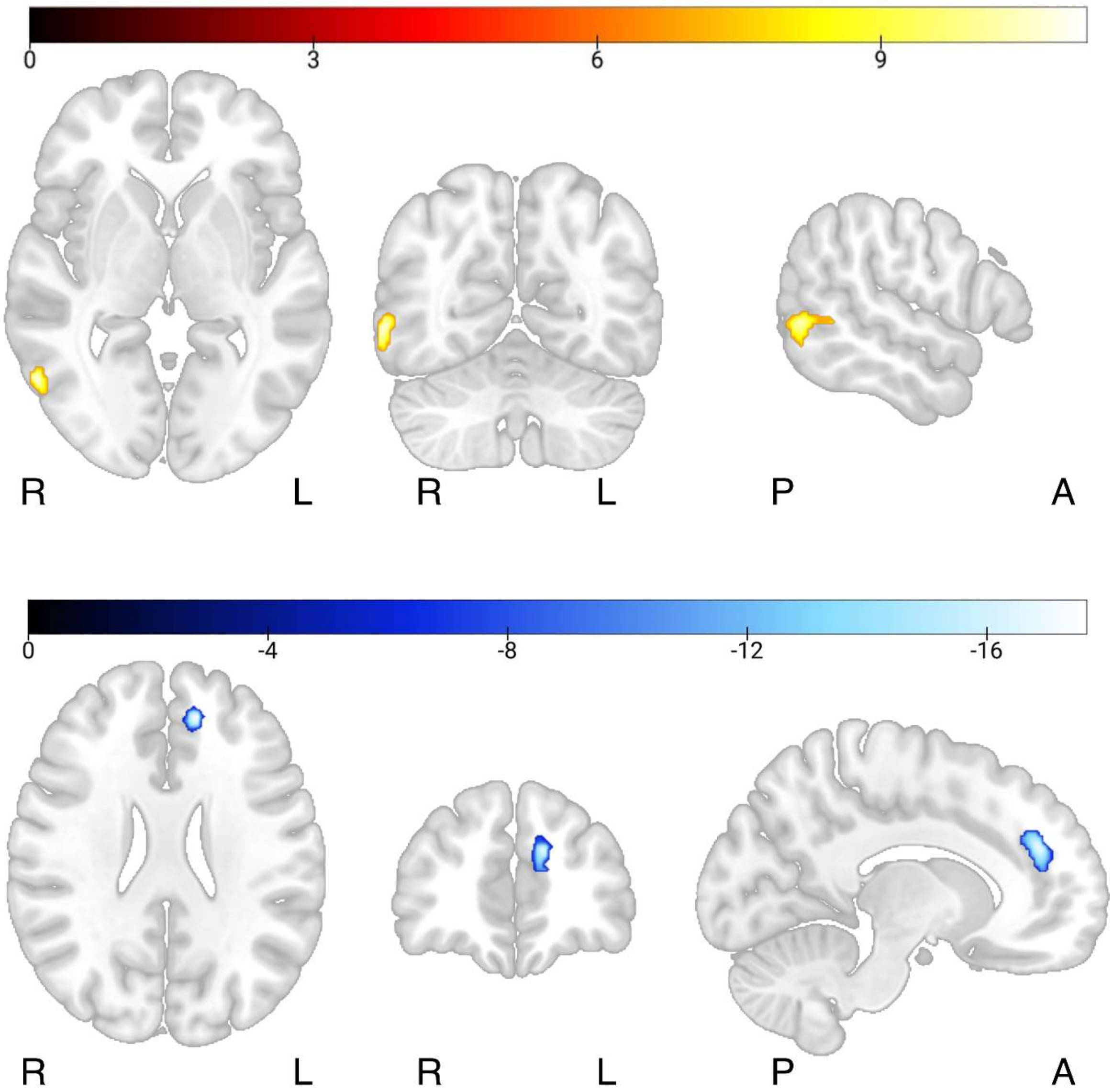

(D) Presence of coating of pharyngeal wall

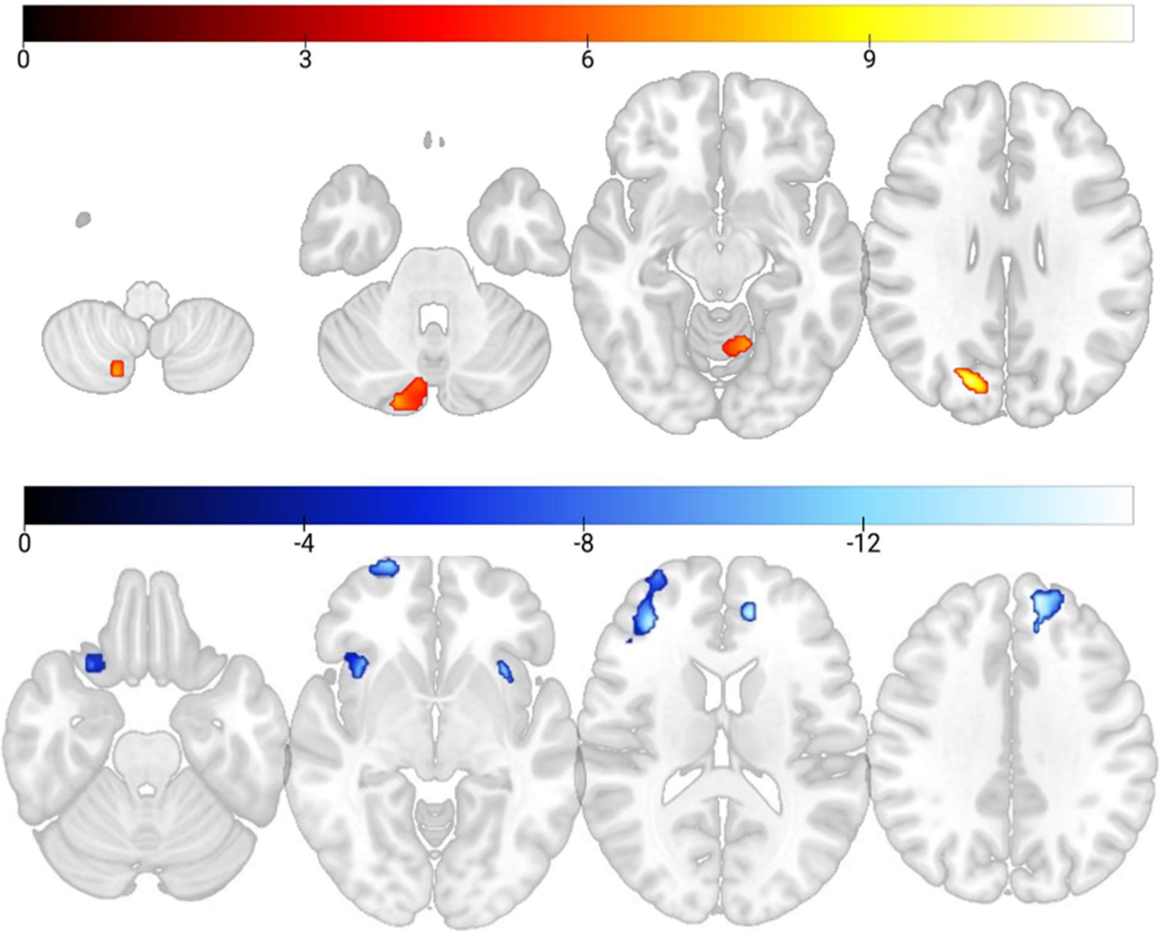

(E) Delayed pharyngeal transit time

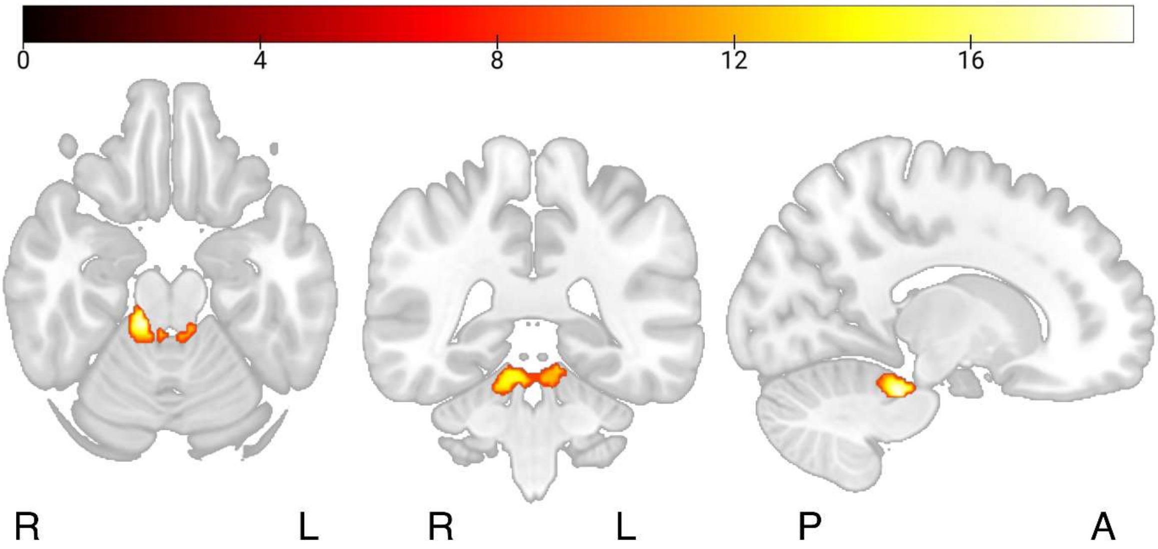

(F) Presence of food aspiration into airways

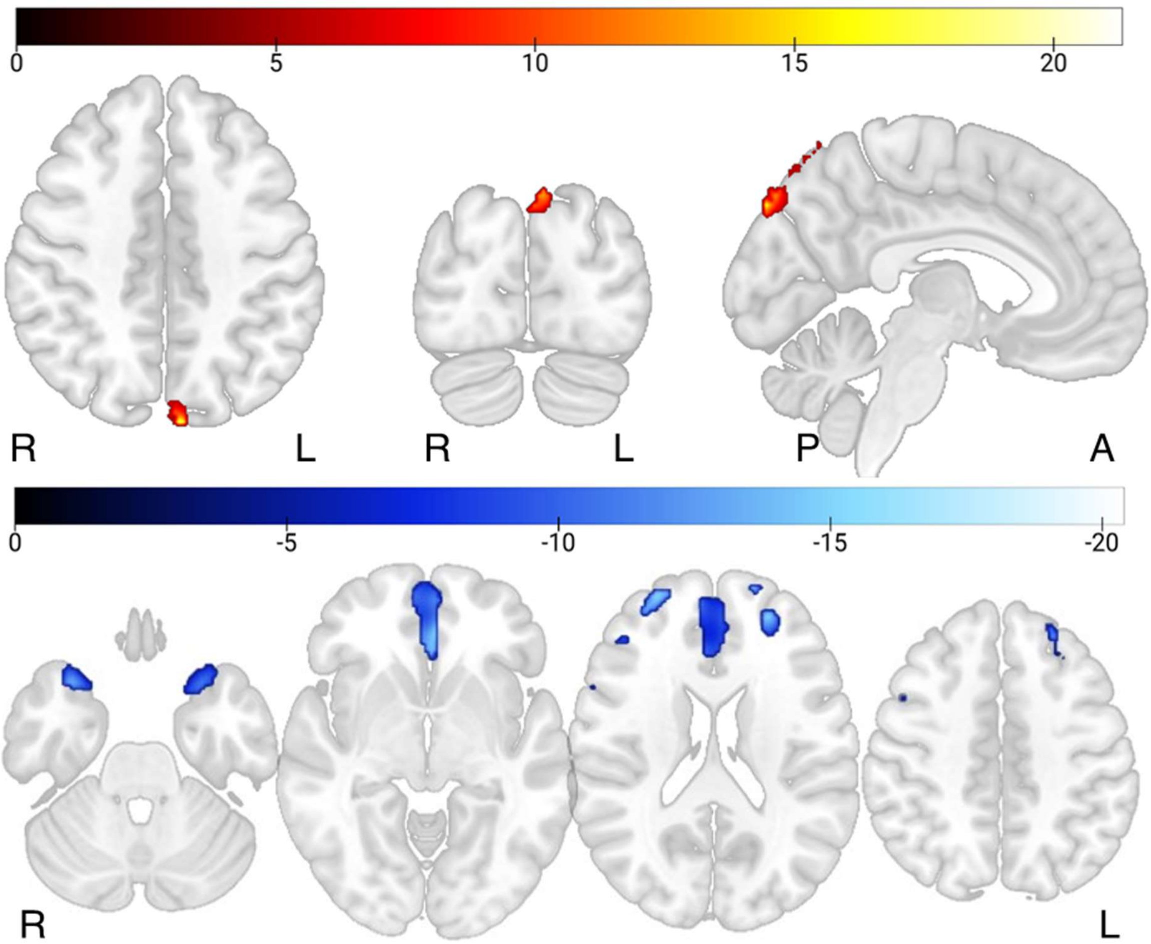

Supplementary Figure 3. Statistical maps showing the metabolic distribution of glucose associated with each dysphagia sub-item in the pharyngeal phase. (**A**) presence of vallecular residue, (**B**) impaired triggering of pharyngeal swallow, (**C**) presence of pyriform sinus residue, (**D**) presence of coating of pharyngeal wall, (**E**) delayed pharyngeal transit time, and (**F**) presence of food aspiration into airways. Only voxels contained in the surviving clusters are displayed. Beta values from the Firth's penalized binary logistic regression were calculated using normalized voxel-wise uptake values and each of the sub-item scores controlling the age and Parkinson's disease duration at the time of the VFSS. The color bar shows beta values: a positive number indicates hypermetabolism, and a negative number indicates hypometabolism. A, anterior; L, left; MNI, Montreal Neurological Institute; P, posterior; R, right; VFSS, videofluoroscopic swallowing study.

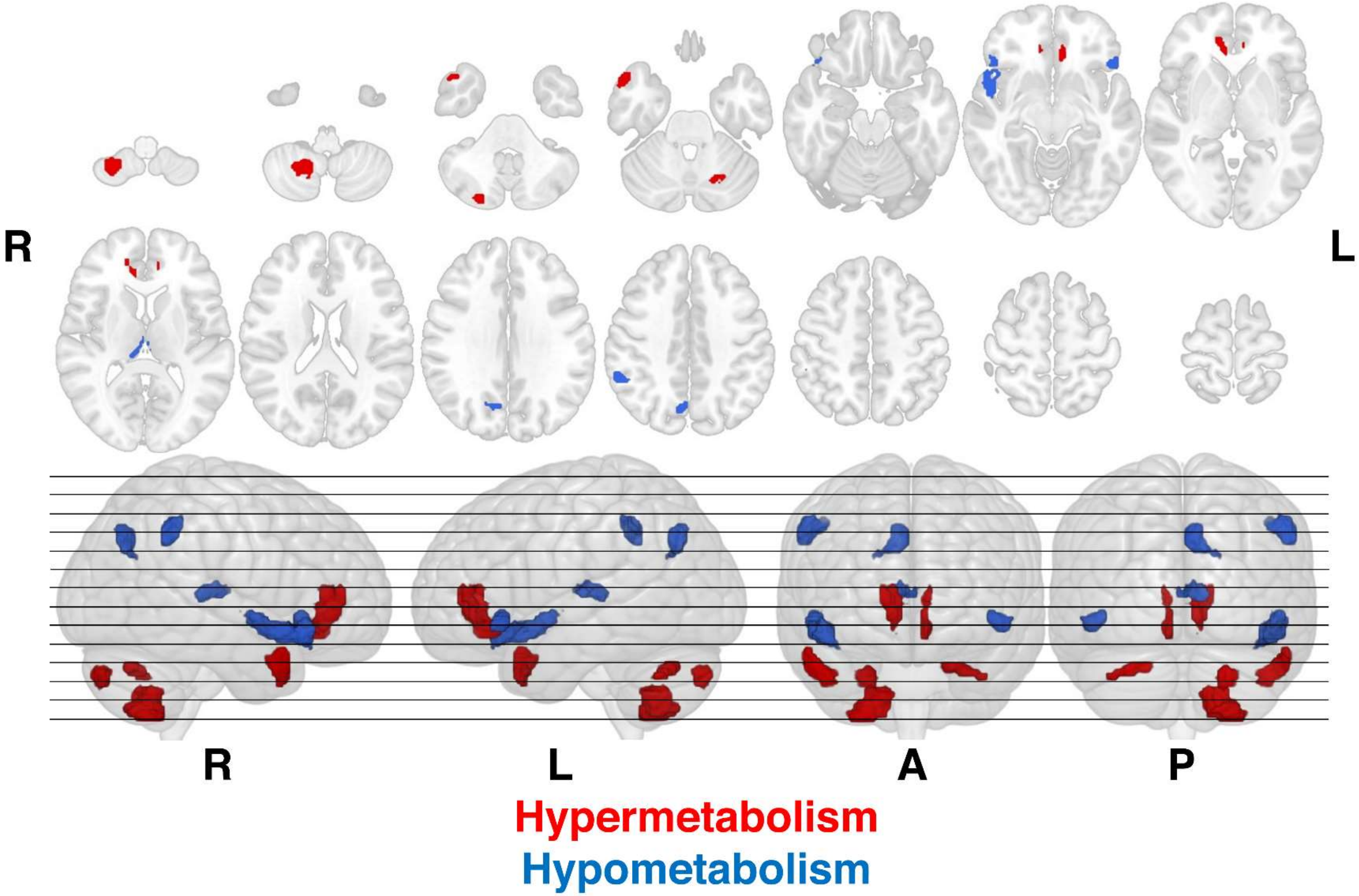

Supplementary Figure 4. Overlay maps showing association of brain glucose metabolism with the oral phase-specific dysphagia. Voxels with values over the threshold (two-tailed voxel-wise *p*-threshold of 0.001, cluster volume of at least 91 voxels) in the multiple regression with adjusted residuals $e_i/(1 - h_i)^{1/2}$ and non-parametric wild bootstrapping (9,999 bootstraps) are shown. The age and Parkinson's disease duration at the time of the VFSS were input as nuisance regressors. The MNI-152 z-coordinates of each row of the slices were -60 -50 -40 -30 -20 -10 0; 10 20 30 40 50 60 70 in ventro-dorsal orders, respectively. Abbreviations: A, anterior; L, left; MNI, Montreal Neurological Institute; P, posterior; R, right; VFSS, videofluoroscopic swallowing study.

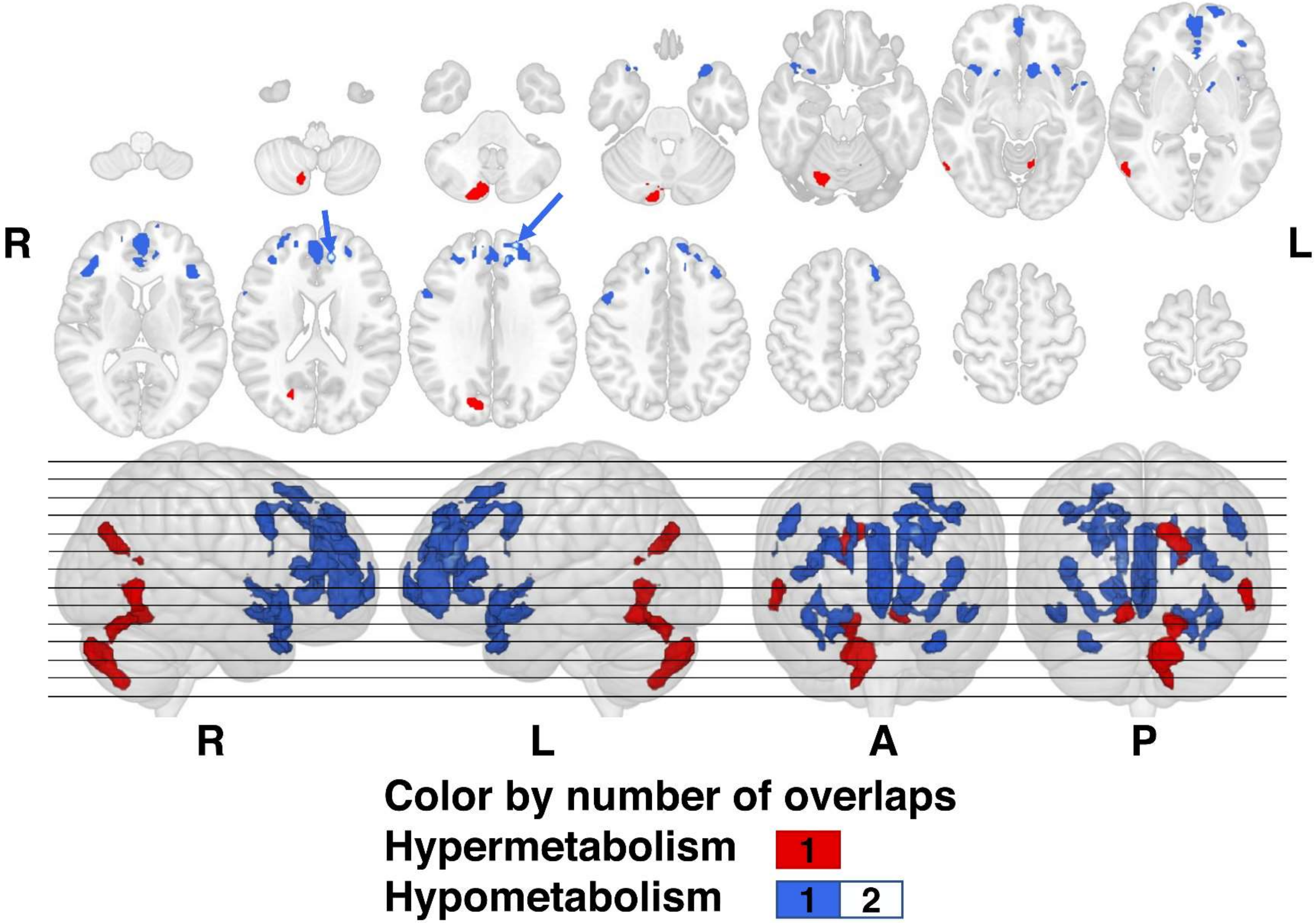

Supplementary Figure 5. Overlay maps showing association of brain glucose metabolism with the pharyngeal phase-specific dysphagia. Integers in the colorbar indicates the phase-specific numbers of overlaps of the clusters surviving the threshold (two-tailed voxel-wise *p*-threshold of 0.001, cluster volume of at least 91 voxels) in the multiple regression with adjusted residuals $e_i/(1 - h_i)^{1/2}$ and non-parametric wild bootstrapping (9,999 bootstraps). The age and Parkinson's disease duration at the time of the VFSS were input as nuisance regressors. The MNI-152 z-coordinates of each row of the slices were -60 -50 -40 -30 -20 -10 0; 10 20 30

40 50 60 70 in ventro-dorsal orders, respectively. Abbreviations: A, anterior; L, left; MNI, Montreal Neurological Institute; P, posterior; R, right; VFSS, videofluoroscopic swallowing study.
